# Symmetric Dopant‐Free Si Solar Cells Enabled by TiO_x_ Nanolayers: An In‐Depth Study on Bipolar Carrier Selectivity

**DOI:** 10.1002/advs.202410179

**Published:** 2024-11-28

**Authors:** Takuya Matsui, Shohei Fukaya, Shona McNab, James McQueen, Kazuhiro Gotoh, Hitoshi Sai, Noritaka Usami, Ruy Sebastian Bonilla

**Affiliations:** ^1^ Renewable Energy Research Center National Institute of Advanced Industrial Science and Technology (AIST) 1‐1‐1 Umezono Tsukuba Ibaraki 305‐8568 Japan; ^2^ Graduate School of Engineering Nagoya University Furo‐cho, Chikusa‐ku Nagoya 464‐8603 Japan; ^3^ Department of Materials University of Oxford Parks Rd Oxford OX1 3PH UK; ^4^ Present address: School of Photovoltaic and Renewable Energy Engineering University of New South Wales Sydney 2052 Australia; ^5^ Present address: Graduate School of Science and Technology Niigata University Niigata 950‐2181 Japan

**Keywords:** atomic layer deposition, carrier selectivity, passivating contact, silicon, solar cell, titanium oxide

## Abstract

High‐efficiency solar cells require two contact structures, engineered for efficient extraction of photogenerated holes and electrons at the respective electrodes. Herein, crystalline Si solar cell featuring hole‐ and electron‐selective passivating contacts composed entirely of a single material, amorphous titanium oxide (TiO_x_), without extrinsic doping is demonstrated. The hole/electron selectivity of the TiO_x_ layers (≈5 nm) is tailored by the oxidation process and the choice of Ti precursor in the atomic layer deposition (ALD). Ex situ and in situ X‐ray photoelectron spectroscopy measurements elucidate that the hole‐selective TiO_x_ induces significant band bending in the Si (Φ≈0.7 eV), generating a *p*‐type inversion layer in the *n*‐Si absorber. The electron‐selective TiO_x_ induces a smaller band bending of Φ<0.35 eV. This clarifies that the bipolar carrier selectivity of TiO_x_ is associated with the different amount of negative fixed charges generated during the ALD process, depending on the choice of Ti precursor and oxidant. In addition, the growth of a hydrogen‐containing SiO_y_ nanolayer (≈1‐1.5 nm) at the Si/TiO_x_ interface during postdeposition oxidation is crucial for providing chemical passivation in both types of TiO_x_. These findings pave the way for a deeper understanding of the charge generation mechanism and chemistry at the Si/metal oxide interfaces.

## Graphical abstract

A symmetric dopant‐free crystalline Si solar cell composed entirely of a single material, amorphous TiO_x_ deposited at the front and the rear, is demonstrated. Ex situ and in situ X‐ray photoelectron spectroscopy measurements revealed that the bipolar carrier selectivity of TiO_x_ is associated with the different amount of negative fixed charge generated during the nanolayer growth in atomic layer deposition.

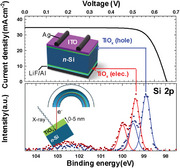

## 1 Introduction

Surface passivation is one of the core technologies in Si photovoltaics because the charge carrier recombination at the electrically‐active Si surface is a major power loss mechanism. When employing high‐quality long‐carrier‐lifetime monocrystalline Si wafers, surface recombination becomes the limiting factor in achieving power conversion efficiencies >20%. Dielectric insulators such as SiO_2_, Al_2_O_3_, and Si_3_N_4_ are the key passivation materials used in industrial Si solar cells, including the passivated emitter rear cell (PERC)^[^ ^1^, ^2^ ^]^ and the tunnel oxide passivated contact (TOPCon)^[^ ^3^, ^4^ ^]^ architectures, whose technologies dominate the current photovoltaic market.^[^ ^5^ ^]^ The passivation is based on two mechanisms: one is the chemical passivation due to the termination of Si dangling bonds by creating chemical bonds between Si and the deposited dielectric layer. Another is field‐effect passivation, which uses the presence of fixed charges at the Si/dielectric interface to minimize the concentration of one type of charge carriers at the Si surface, leading to an effective mitigation of surface recombination. However, due to the electrical insulative nature of these dielectric materials, a local Si‐metal contact needs to be formed through the dielectric layer to extract photogenerated charge carriers out of the Si. In such a metalized area, the surface defects remain electrically active, limiting the power conversion efficiency.

Recently, advanced schemes that enable surface passivation and carrier collection without a metal‐Si interface have been developed and termed “passivating contacts”.^[^ ^6^ ^]^ Hydrogenated amorphous Si (a‐Si:H) layers stacks provide an excellent passivating contact on crystalline Si, known as the silicon heterojunction (SHJ) architecture,^[^ ^7^ ^]^ leading to one of the highest Si solar cell efficiencies of 26.8% in a top–bottom contacted device structure.^[^ ^8^ ^]^ Currently, however, the global market share of the SHJ technology is still below 10%^[^ ^5^ ^]^ due to the higher capital expenditure compared to the homojunction Si solar cell architectures, including PERC and TOPCon.

Recently, dopant‐free semiconducting metal compounds, such as MgO_x_,^[^ ^9^ ^]^ TaN_x_,^[^ ^10^ ^]^ TiN_x_,^[^ ^11^ ^]^ TiN_x_O_y_,^[^ ^12^ ^]^ MoO_x_,^[^ ^13^ ^]^ and V_2_O_x_^[^ ^14^ ^]^ have attracted increased attention as they have shown the potential to deliver equivalent passivating contact functionality to the SHJ.^[^ ^6^, ^15^ ^]^ They can potentially replace the conventional dielectric insulators used in homojunction solar cells leveraging high efficiency and low manufacturing cost. Among the various materials reported so far, titanium oxide (TiO_x_ or TiO_2_) exhibits a unique feature. Classically, TiO_x_ is known to act as an electron‐selective passivating contact in Si solar cells, demonstrating efficiency above 22%.^[^ ^16^, ^17^, ^18^ ^]^ The electron selectivity of the TiO_x_ has been ascribed to an asymmetric band offset at the Si/TiO_x_ interface, where the small conduction band offset (≈0.1 eV) allows electron conduction while the large valence band offset (>2 eV) inhibits the hole conduction. Contrary to this general understanding, TiO_x_ has been proven to act as a “hole selective” passivating contact and applied to *n*‐ and *p*‐Si solar cells leading to efficiencies >20%.^[^ ^19^, ^20^ ^]^ This unusual hole selectivity is attributed to the negative fixed charge present at the Si/TiO_x_ interface, in analogue to the charging mechanisms at the Si/Al_2_O_3_ interface.^[^ ^21^, ^22^, ^23^, ^24^, ^25^ ^]^ It has been demonstrated that the carrier selectivity of the TiO_x_ layer can be tuned by the different atomic layer deposition (ALD) processes using plasma‐ and thermal‐oxidation reactions.^[^ ^19^, ^26^ ^]^ This enables the exploitation of a single material system that can behave both as an electron or a hole selective contact in solar cells. We call this functionality bipolar carrier selectivity. Despite extensive work in TiO_x_ thin film materials, the physical and chemical mechanisms that enable bipolar carrier selectivity with respect to Si are not yet fully understood, especially in relation to the TiO_x_ deposition processes.

In this study, we investigate the properties of the TiO_x_ layers prepared by thermal‐ and plasma‐ALD processes using different Ti precursors for use in passivating contacts. We have successfully synthesized TiO_x_ layers that act either electron‐ or hole‐selective contacts, while providing moderate Si surface passivation. This allows us to fabricate a novel symmetrically TiO_x_‐contacted Si solar cell structure with ≈19% efficiency. The nature of bipolar carrier selectivity in a single TiO_x_ material is investigated using multiple characterization techniques, and it is revealed that the carrier selectivity is primarily associated with the chemical reaction of Ti precursors and oxidant during the growth of TiO_x_ on Si.

## 2 Results

### 2.1 Passivation and Contact Properties of Differently Prepared TiO_x_ Layers

**Figure 1:**
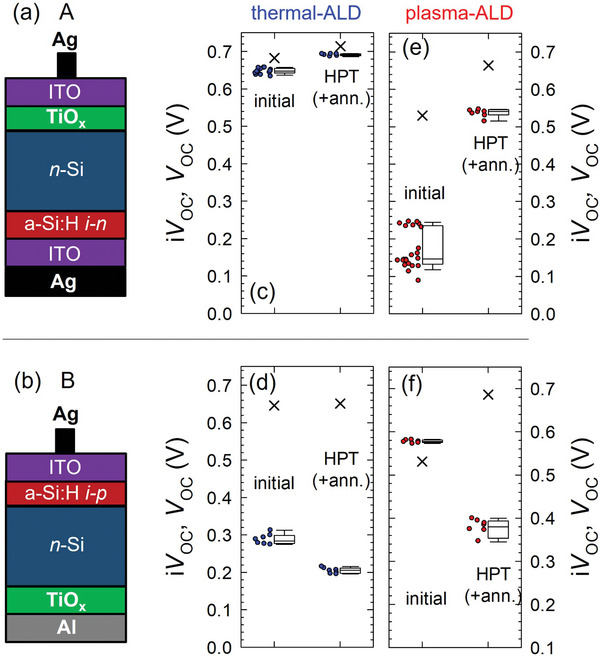
a, b) Test solar cell structures of (a) device A and (b) device B that have *n*‐ and *p*‐doped a‐Si:H layers for evaluating hole and electron selectivity of the TiO_x_ layers, respectively. (c–f): i*V*_OC_ (crosses) and *V*_OC_ (circles) of the test solar cells with TiO_x_ layers made by c, d) thermal‐ALD (TTIP) and e, f) plasma‐ALD (TTIP) in the initial state and after hydrogen plasma treatment (HPT) followed by thermal annealing at 180 °C. The results obtained using the devices A and B correspond to (c, e) and (d, f), respectively.

It has been previously shown that carrier selectivity of TiO_x_ can be tuned by oxidizing the Ti precursor (titanium tetraisopropoxide: TTIP) with either H_2_O vapor (thermal‐ALD) or O_2_ plasma (plasma‐ALD) during each ALD growth cycle.^[^ ^26^ ^]^ To prove the opposite carrier selectivity of TiO_x_ in an actual device structure, we fabricated test solar cells A and B, as shown in **Figure** **1**. We deposited either thermal‐ or plasma‐ALD TiO_x_ on one side of the *n*‐Si (100) wafer. The other side of Si was capped with a stack of undoped/doped a‐Si:H layers. By using *n*‐ and *p*‐doped a‐Si:H layers in devices A and B, respectively, device A can be used to test the hole selectivity of the TiO_x_ layer, and device B to test its electron selectivity. The passivation of the Si/TiO_x_ interface was evaluated by measuring the implied open‐circuit voltage (i*V*_OC_) of the solar cell precursors using a quasi‐steady state photoconductance (QSSPC) setup prior to front and rear metallization. The carrier selectivity of the TiO_x_ layers was characterized by the deviation of the measured open‐circuit voltage (*V*_OC_) of the finished solar cells from its i*V*_OC_.^[^ ^27^, ^28^ ^]^ As shown in Figure 1c,d, thermal‐ALD TiO_x_ provides both relatively high i*V*_OC_ (>0.7 V) and *V*_OC_ (0.65‐0.7V) in the case of device A, whereas it results in a significantly lower *V*_OC_ (<0.3 V) than i*V*_OC_ (>0.7 V) in the case of device B. The current‐density voltage (*J−V*) curves of these test devices are included in Figure S1 (Supporting Information). On the other hand, plasma‐ALD TiO_x_ shows the opposite tendency (Figure 1e,f). A smaller i*V*_OC_ *−V*_OC_ deviation is observed for device B than for device A. These results clearly show that thermal‐ALD TiO_x_ exhibits a hole‐selective nature, while the plasma‐ALD TiO_x_ exhibits an electron‐selective nature.

In Figure 1c–f, the results after applying a hydrogen plasma treatment (HPT) and thermal anneal on the TiO_x_ are included. The HPT effectively improves Si/TiO_x_ interface passivation (i*V*_OC_) for both test structures, and it improves *V*_OC_ of device A. However, it degrades *V*_OC_ of device B, indicating that HPT is prone to increase the hole‐selective nature of the TiO_x_.

The results in Figure 1 show that a major issue in applying plasma‐ALD TiO_x_ to Si is the poor passivation quality (i*V*_OC_ ≈0.55 V), especially when compared to the thermal‐ALD TiO_x_ or the HPT treated samples. This is mainly attributed to the lack of hydrogen at the Si/TiO_x_ interface.^[^ ^19^ ^]^ To demonstrate an efficient TiO_x_ electron‐selective contact, we replace the plasma‐ALD TiO_x_ by a thermal‐ALD TiO_x_ using a different Ti precursor tetrakis(dimethylamino)titanium (TDMAT), which has been previously used in the development of electron‐selective contacts.^[^ ^29^, ^30^, ^31^ ^]^

**Figure 2:**
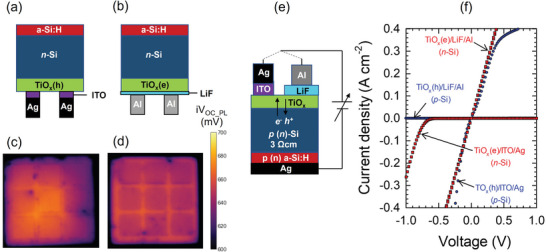
a, b) Sample structures for assessing the passivation properties at the (a) Si/TiO_x_(h) (TTIP) and (b) Si/TiO_x_(e) (TDMAT) interfaces. c, d) i*V*_OC_PL_ maps of the corresponding sample structures that have (c) Si/TiO_x_(h) and (d) Si/TiO_x_(e) interfaces measured by PL imaging whose signal intensity was calibrated by QSSPC measurement. Nine capping electrode pads (1 cm × 1 cm) of (c) ITO/Ag and (d) Al were formed by sputtering and thermal evaporation, respectively, through a metal shadow mask. e) Sample structure for contact resistivity measurement and f) the corresponding dark *J−V* characteristics with various combinations of TiO_x_ (TiO_x_(h) and TiO_x_(e)) and capping electrode materials (ITO/Ag and LiF/Al).

First, we studied the passivation quality and carrier selectivity of the TiO_x_ layers prepared by thermal‐ALD using TTIP and TDMAT precursors. Hereafter, we term these materials as TiO_x_(h) and TiO_x_(e), respectively, to indicate hole or electron selectivity. The passivation quality of the Si/TiO_x_(h) and Si/TiO_x_(e) interfaces was assessed by the photoluminescence (PL) measurement on sample structures shown in **Figure** **2**a,b, respectively. The PL intensity was calibrated by the QSSPC measurement on the same structure before metallization,^[^ ^30^ ^]^ enabling the estimation of the spatial i*V*_OC_ (i*V*_OC_PL_) in an actual device structure even after metallization is performed on the TiO_x_ layer (Figure S2, Supporting Information). As depicted in the sample structures, the rear TiO_x_ layers (5 cm × 5 cm) were partially coated with nine electrode pads (1 cm × 1 cm). We used sputtered ITO/Ag^[^ ^19^ ^]^ and evaporated Al^[^ ^16^, ^17^, ^18^, ^29^, ^30^, ^31^, ^32^ ^]^ as capping electrodes which are typically used for hole and electron contacts, respectively. For the Si/TiO_x_(e) sample, an evaporated LiF buffer layer was deposited over the whole area of the TiO_x_ prior to Al capping. The results show that both the TiO_x_(h) and TiO_x_(e) layers provide high i*V*_OC_PL_ of 680–700 mV in the unmetallized area, whereas it is lowered by more than 30 mV when capped with Al (Figure 2c,d). We attribute this Al‐induced passivation degradation to two possible factors: one is the decrease of field‐effect passivation by capping the TiO_x_ layer with a low work‐function metal.^[^ ^20^ ^]^ The other possibility is the chemical reaction between the TiO_x_ and Al contact.^[^ ^33^ ^]^ We highlight that the passivation degradation becomes even greater (Δi*V*_OC_PL_ >100 mV) in the absence of LiF buffer layer (not shown).

Next, the electrical properties of these TiO_x_ layers were characterized by performing the contact resistivity (*ρ*_c_) measurement on sample structures prepared using *p*‐Si and *n*‐Si substrates as shown in Figure 2e. In Figure 2f, the measured dark *J−V* characteristics are shown. An ohmic behavior is obtained when depositing TiO_x_(h) on *p*‐Si and TiO_x_(e) on *n*‐Si as seen from Figure 2f. Note that the right capping electrodes (high work‐function ITO/Ag for TiO_x_(h), and low work‐function LiF/Al for TiO_x_(e)) are necessary to achieve optimal carrier collection. Although the *J−V* curve of the *p*‐Si/TiO_x_(h)/ITO/Ag layer stack slightly deviates from ideal ohmic behavior in the forward voltage applied regime, a *ρ*_c_ as low as ≈5 × 10^2^ mΩ cm^2^ is obtained in the reverse voltage regime, where the holes are extracted from Si to TiO_x_ in agreement with the transport direction under solar cell operation. From these electrical properties, it is clear that the TiO_x_(h) synthesized from TTIP exhibits a hole‐selective nature while the TiO_x_(e) from TDMAT exhibits an electron‐selective nature. As reported previously,^[^ ^20^, ^34^ ^]^ the combination of TiO_x_(h) (TiO_x_(e)) and high (low) work‐function capping electrodes is crucial to attain low contact resistivity. From Figure 2f, it is also clear that improper combinations of contact material and capping electrode (i.e., TiO_x_(h) with LiF/Al, or TiO_x_(e) with ITO/Ag) result in a rectifying *J−V* behavior and an increase in contact resistivity by several orders of magnitude. This indicates that carrier selectivity of the TiO_x_ layer cannot be converted by simply changing the capping electrode material. It should be added that the thickness of the TiO_x_ layer is also an optimization parameter for solar cell performance. However, a previous study showed that it does not significantly alter the carrier selectivity within the thickness range from 3 to 30 nm.^[^ ^26^ ^]^ While thinning TiO_x_ layer to ≈1–1.5 nm and using a different capping electrode could convert the carrier selectivity, we observed a marked passivation degradation in both TiO_x_(h) and TiO_x_(e) for such low thicknesses (not shown). Thus, in this work, we maintained the TiO_x_ thickness at ≈5 nm, as it is optimal for solar cell performance.

### 2.2 Symmetrically TiO_x_‐Contacted Si Solar Cells

**Figure 3:**
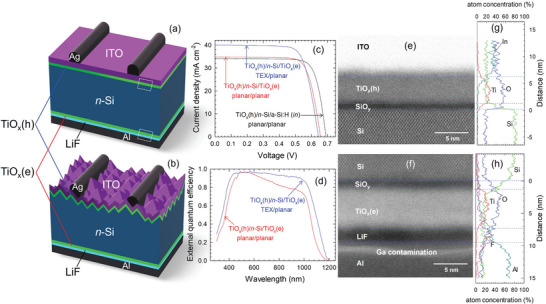
a,b) Schematic illustrations of the symmetrically TiO_x_‐contacted solar cells using (a) double sided planar (DSP) and (b) front‐textured rear‐planar (FT_RP) *n*‐Si wafers. c) *J−V* curves and d) external quantum efficiency (EQE) spectra of the solar cells. In (c), a *J−V* curve of a reference DSP cell that has a TiO_x_(h) layer at the front and a‐Si:H *in* layers at the rear is shown for comparison. e, f) HAADF‐STEM cross‐sectional images of the DSP solar cell taken at the (e) front side (TiO_x_(h)) and (f) the rear side (TiO_x_(e)), and g, h) the corresponding EDX elemental line scans ((g): front and (h): rear). High Si and O concentrations appeared in the non‐Si and non‐oxide layers (≈20 at.%), respectively, are probably caused by the relatively high background contamination level inside the STEM‐EDX setup.

Following the above characterization of the passivation and contact properties of the TiO_x_ layers, we fabricated solar cells with a symmetrically TiO_x_‐contacted structure. **Figure** **3**a–d shows the device structures and the corresponding solar cell performance of the *n*‐Si solar cells. TiO_x_(h) was deposited on the front illumination side of *n*‐Si as it allows capping with a transparent ITO electrode. Two types of Si substrates were employed: a double‐sided planar Si (100) (DSP‐Si) and a front‐textured rear‐planar Si (FT_RP‐Si), leading to conversion efficiencies of 17.4% and 19.1%, respectively. The results successfully demonstrate an operation of the world's first Si solar cells featuring dopant‐free hole‐ and electron‐selective passivating contacts composed entirely of a single material. However, the *V*_OC_ of both DSP‐Si and FT_RP‐Si (≈0.65 V) are somewhat lower than the *V*_OC_ ≈0.69 V of a reference asymmetrically contacted DSP cell (TiO_x_(h) front hole contact and *i‐n* a‐Si:H rear electron contact) shown in the same Figure. Since the i*V*_OC_PL_ measured on the device precursors before the Al metallization was ≈0.7 V, it appears that the decrease in *V*_OC_ occurs when the Al electrode is formed on top of the TiO_x_(e)/LiF, as shown in Figure 2d. Although the LiF buffer layer is found to greatly mitigate the Al‐induced passivation degradation (not shown in this paper), the *V*_OC_ loss of ≈20 meV still remains. As a proof of a concept, a test device was prepared with a local contact design in which the Al electrode only partially covers the rear, showing an improved *V*_OC_ of 0.673 V. This result indicates that there is room for further improvement in device performance.

Figure 3e,f show high‐angle annular dark‐field scanning transmission electron microscope (HAADF‐STEM) images of the cross‐section of the 17.4% efficient DSP solar cell highlighting the front Si/TiO_x_(h) and the rear Si/TiO_x_(e) interfaces (rectangular areas of Figure 3a), respectively. The corresponding compositional profiles obtained by energy dispersive X‐ray (EDX) analysis are shown in Figure 3g,h. Both TEM images show atomically abrupt interfaces between adjacent layers, except at the LiF/Al interface (Figure 3f) due to a contamination issue caused by a Ga‐ion beam milling process for TEM sample preparation. No fringe pattern is visible in both TiO_x_ layers, indicating that both TiO_x_(h) and TiO_x_(e) films are predominantly amorphous. Similar to the previous studies,^[^ ^19^, ^35^ ^]^ a silicon oxide (SiO_y_) nanolayer that contains a slight amount of Ti is created at both the Si/TiO_x_(h) and Si/TiO_x_(e) interfaces. It is found that the thickness of this interface layer is slightly thinner for the Si/TiO_x_(h) (≈1.0 nm) than that for the Si/TiO_x_(e) (≈1.5 nm). In both the TiO_x_(h) and TiO_x_(e) bulk layers, O/Ti compositional ratio nearly matches the stoichiometric composition (O/Ti = 2.0), consistent with the compositional analysis done by Rutherford backscattering (RBS) spectroscopy on TiO_x_ layers deposited on Si without metal/TCO capping (Figure S3 and Table S1, Supporting Information). From this characterization, it is not possible to associate the different carrier selective nature of the TiO_x_(h) and TiO_x_(e) layers with their structural or compositional properties.

### 2.3 Si/TiO_x_ Interface Characterizations

**Figure 4:**
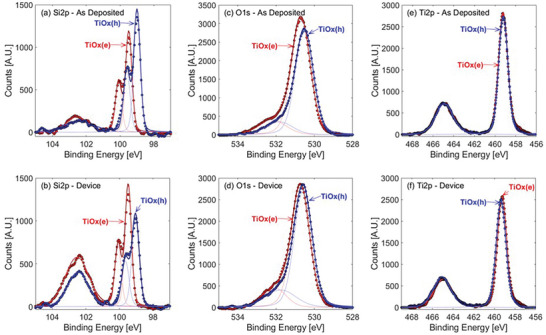
XPS spectra measured ex situ on *n*‐Si (100)/TiO_x_ (≈5 nm) samples with TiO_x_(h) (blue circles, TTIP) and TiO_x_(e) (red circles, TDMAT) layers. Lines represent component peak‐fitting of XPS spectra. a, b) Si 2p, c, d) O 1s and e, f) Ti 2p. The XPS data were taken in the (a),(c),(e) as deposited state and (b),(d),(f) after the same postdeposition treatment (PDT) as used for solar cell fabrication (TiO_x_(e): annealing at 180 °C in low vacuum, TiO_x_(h): hydrogen plasma treatment followed by annealing at 180 °C in low vacuum).

To gain insight into the mechanisms by which a single dopant‐free material acts as bipolar carrier selective contact with respect to Si, we applied X‐ray photoelectron spectroscopy (XPS) to probe the Si/TiO_x_ interface properties. **Figure** **4** shows the XPS core level spectra of Si 2p, O 1s, and Ti 2p measured for the TiO_x_(h) and TiO_x_(e) layers deposited on an H‐terminated *n*‐Si (100). The thickness of these TiO_x_ layers is ≈5 nm, which is equivalent to that used in solar cells, and thin enough to detect photoelectrons from the Si near‐surface. These ex situ XPS measurements were carried out in an as‐deposited state and after applying the same postdeposition treatments (PDT) as used in the solar cell fabrication (i.e., annealing for TiO_x_(e) and HPT followed by annealing for TiO_x_(h)). The lowest pass energy (6.5 eV) available in the electron energy analyzer was used to obtain the highest spectral resolution. As shown in Figure 4a,b, a clear difference is found in the Si 2p spectra between the two TiO_x_ samples. The energy position of the Si 2p peak (a set of doublet peaks of Si 2p_1/2_ and Si 2p_3/2_) for the TiO_x_(h) sample shifts toward the lower binding energy by ≈0.45 eV with respect to that of TiO_x_(e). Since these TiO_x_ act as passivation layers, the Fermi level at the Si/TiO_x_ interface could not be strongly pinned by surface defects. Thus, the Si 2p peak energy position reflects the Fermi level position in the *n*‐Si near the Si/TiO_x_ interface.^[^ ^36^, ^37^ ^]^ The shift of Si 2p peak toward the lower binding energy observed for the TiO_x_(h) provides the evidence that the Fermi level is shifted to a deeper energy toward the valence band. This experimental fact is in line with the previous findings that TiO_x_(h) contains negative fixed charges in the order of >10^12^ cm^−2^.^[^ ^20^ ^]^ From the results shown in Figure 4a,b, it is found that Si 2p peak energy does not change before and after PDT. This manifests that the Fermi‐level shift resulting from fixed charge generation is not altered by PDT such as annealing and hydrogenation. On the other hand, a broader and more intense peak associated with the Si‐O bonds is observed at binding energies of ≈102.6 eV for samples that underwent PDT, indicating that oxidation takes place at the Si/TiO_x_ interface due to the oxygen diffusion through the TiO_x_ layer during annealing in PDT.

Compared to the TiO_x_(h), the TiO_x_(e) has a higher Si‐O peak intensity, particularly after PDT. This reflects the slightly thicker SiO_y_ interfacial layers for TiO_x_(e) than for TiO_x_(h) as shown in the TEM cross‐sectional images (Figure 3e,f). The less dense TiO_x_(e) microstructure compared to the TiO_x_(h) is evidenced by RBS measurements (Table S1, Supporting Information), providing a possible reason for the enhanced oxygen diffusion in TiO_x_(e) during the annealing. The fact that passivation of the Si/TiO_x_ interface is greatly enhanced only when annealing in an oxygen (or water) containing atmosphere, indicates that the interfacial SiO_y_ layer formed during annealing is crucial to a high‐quality chemical interface (here we use a low vacuum oven, ≈10 Pa of air at 180 °C). However, it should be noted that the presence of hydrogen at the Si/TiO_x_ interface (>1 at.%) was found to play an important role in passivation.^[^ ^19^ ^]^ In fact, elastic recoil detection analysis (ERDA), in combination with RBS measurements, confirmed the presence of 1.4 at.% hydrogen in TiO_x_(h) and 9.9 at.% in TiO_x_(e) in the as‐deposited state (Supporting Information Table S1, Supporting Information). H_2_O used in the thermal‐ALD process acts as a source of hydrogen, as no hydrogen was detected in plasma‐ALD TiO_x_ layer.^[^ ^19^ ^]^

In Figure 4c,d, a less pronounced but similar peak shift is seen in the O 1s spectra of the TiO_x_(h). However, the peak shift of O 1s is much smaller than that of Si 2p because the O 1s signal reflects mostly the chemical state in the TiO_x_ bulk layer. As will be shown later, the O 1s peak position depends on the thickness of the TiO_x_ layer. Meanwhile, the Ti 2p spectra for the TiO_x_(h) and TiO_x_(e) samples are almost completely overlapped (Figure 4e,f), indicating no marked difference in the chemical properties between the bulk of these two TiO_x_(h) and TiO_x_(e) layers.

**Figure 5:**
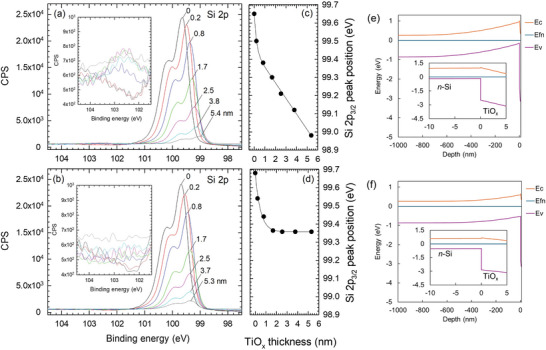
a, b) XPS Si 2p spectra measured in situ during the ALD process for (a) TiO_x_(h) (TTIP) and (b) TiO_x_(e) (TDMAT), and c, d) the corresponding peak energy positions with different TiO_x_ thicknesses (*t*_TiOx_). The *t*_TiOx_ shown here was determined using the final layer thickness (≈5 nm) divided by the number of ALD cycles. Depositions and measurements were done by transferring the Si substrate between ALD and XPS chambers under high vacuum (<10^−5^ Pa). Insets in (a) and (b) are the close‐up views at binding energies ≈102–104 eV where the Si‐O bonding signal appears. e, f) Band diagrams of (e) *n*‐Si/TiO_x_(h) and (f) *n*‐Si/TiO_x_(e) obtained by a SCAPS finite element device simulation. *E*_c_ and *E*_v_ denote energy levels of conduction band and valence band, respectively. *E*_fn_ is the Fermi level. The interface charges of (e) *Q*_f_ = ‐1.1 × 10^13^ q cm^−2^ and (f) *Q*_f_ = ‐5.0 × 10^12^ q cm^−2^ were assumed to achieve band bending of ≈0.7 eV and ≈0.35 eV, respectively, which were estimated by XPS measurements.

The ex situ XPS measurements determine that the band bending induced in *n*‐Si (XPS Si 2p peak shift) by TiO_x_ deposition is not changed by postdeposition treatments such as annealing (oxidation) and hydrogenation. Therefore, the negative fixed charges are likely to be generated during the TiO_x_ deposition. To confirm this, we conducted in situ XPS measurements, alternating between ALD TiO_x_ deposition and XPS analysis without any air break during the sample transfer between the ALD and XPS chambers. **Figure** **5**a,b show the measured Si 2p spectra with various TiO_x_ layer thicknesses (*t*_TiOx_) deposited on *n*‐Si for TiO_x_(h) and TiO_x_(e), respectively. Note that a larger pass energy was used in the in situ XPS measurement (55 eV) than that in the ex situ counterpart (6.5 eV) to minimize the interruption time of the ALD process, resulting in broadening of the Si 2p spectrum. The Si 2p peak intensity attenuates with *t*_TiOx_ due to the lower escape probability of the photoelectrons from the Si. Nevertheless, the Si 2p peak energy can still be evaluated in the same way as in Figure 4a,b.

From these in situ XPS measurements, it is revealed that Si 2p peak shift reflecting the band bending of *n*‐Si already starts after a few cycles of ALD (*t*_TiOx_ ≈ 0.2 nm) and it is enhanced with thickening both the TiO_x_(h) and TiO_x_(e) layers. In Figure 5c,d, the Si 2p peak energy for these samples is plotted as a function of *t*_TiOx_. It appears that the TiO_x_(h) deposition leads to a steep Si2p peak shift until *t*_TiOx_ ≈1 nm, followed by a continuous peak shift at a lower rate with increasing *t*_TiOx_. When *t*_TiOx_ ≈5 nm, which is the same thickness as used in solar cells, the Si 2p peak shift is 0.7 eV with respect to the initial peak energy position of the H‐terminated *n*‐Si (100) surface (*t*_TiOx_ = 0 nm). In the absence of other electrostatic mechanisms, this indicates a surface band bending potential as high as 0.7 V. Similarly, the TiO_x_(e) deposition leads to a Si 2p peak shift of 0.35 eV for *t*_TiOx_ < 1.5 nm, while it is completely saturated with further thickening the layer. At *t*_TiOx_≈ 5 nm, the difference in the peak energy shift between the TiO_x_(h) and TiO_x_(e) is slightly lower for in situ XPS (≈0.35 eV) than for ex situ XPS (≈0.45 eV) (Figure S4, Supporting Information). Although the difference is rather minor, we note that this could be attributed to the use of different ALD systems for ex situ and in situ XPS measurements.

From the XPS spectra in Figure 5a,b, it is also noticeable that the rate of decline in Si 2p peak height against *t*_TiOx_ is similar between the TiO_x_(h) and TiO_x_(e). This indicates that there is no marked difference in the growth and coverage of these TiO_x_ layers on Si surface throughout the ALD process. Another unique feature found in these in situ XPS measurements is the very low Si‐O peak intensity (102.6 eV) during the TiO_x_ deposition, unlike those measured in ex situ XPS (Figure S4, Supporting Information). This provides evidence that the interfacial SiO_y_ layer is predominantly formed after the air break and the following annealing step. This will be further discussed later.

**Figure 6:**
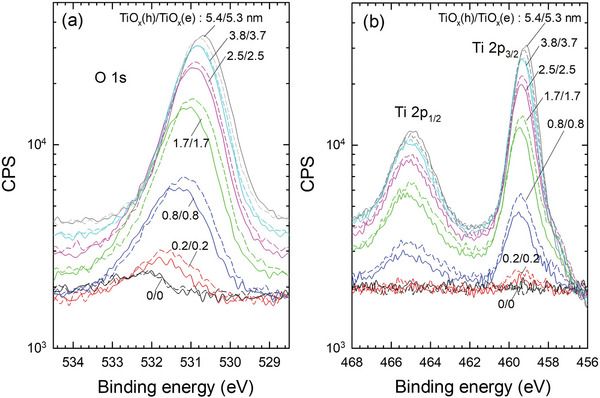
a) O 1s and b) Ti 2p spectra measured by in situ XPS during the ALD process for TiO_x_(h) (solid lines, TTIP) and TiO_x_(e) (dashed lines, TDMAT) with various TiO_x_ thicknesses (*t*_TiOx_). The XPS signal intensity is shown in a logarithmic scale to better present the weak signal of thinner TiO_x_ layers.

The in situ XPS spectra were also acquired on O 1s and Ti 2p (2p_1/2_ and 2p_3/2_) peaks. These are shown in **Figure** **6**, which reveals that, with increasing *t*_TiOx_, these peak energies are shifted from higher binding energies toward those of the stoichiometric TiO_2_. This chemical shift can be attributed to the presence of Si (or SiO_2_) in/under the TiO_x_ layer.^[^ ^38^ ^]^ Compared to the TiO_x_(e), both the O 1s and Ti 2p peaks of TiO_x_(h) are centered at higher binding energies. This indicates that more oxygen atoms are prone to bond to Si, particularly at the beginning of the TiO_x_(h) growth (*t*_TiOx_ < 1.5 nm), in agreement with the fact that more SiO_y_ is grown at the Si/TiO_x_(h) than at the Si/TiO_x_(e) interface, as shown in Figure 5a,b.

Additional characterization via surface photovoltage (SPV) by Kelvin probe (KP) and conductance‐voltage (*G−V*) measurements^[^ ^20^ ^]^ provided further experimental evidence of the different nature of the TiO_x_(h) and TiO_x_(e) materials deposited on Si. The SPV measurement was carried out on an *n*‐Si/TiO_x_ sample structure. The results show that the contact potential deference (CPD) signals of these TiO_x_ layers showed the same sign (Figure S5, Supporting Information), indicating that both the TiO_x_(h) and TiO_x_(e) layers contain negative fixed charges. However, CPD signal is found to be higher for TiO_x_(h) than for the TiO_x_(e). In addition, a *G−V* measurement was done on an *n*‐Si/TiO_x_/Al sample structure. The experimental *G−V* curves as well as their model fitting described in ref. [20] indicate that TiO_x_(h) has a larger negative fixed charge concentration (≈3 × 10^12^ cm^−2^) than that of TiO_x_(e) (<2 × 10^12^ cm^−2^) (Figure S6, Supporting Information). These SPV and *G−V* measurement results agree with the XPS analysis shown above.

## 3 Discussion

Based on the above characterization, it can be concluded that a large amount of negative fixed charge (>10^12^ cm^−2^) is generated at the initial stage of deposition (*t*_TiOx_ ≈1 nm) in both TiO_x_(h) and TiO_x_(e). For TiO_x_(h), the Si band bending as high as 0.7 eV, which is greater than half of the Si bandgap, induces a hole‐rich inversion layer leading to preferential hole transport. Device simulations (Figure 5e,f; Figure S7, Supporting Information) show that the formed *n*‐Si/TiO_x_(h) interface has a negative charge concentration of the order of 1 × 10^13^ cm^−2^ to induce a ≈0.7 eV band bending in *n*‐Si. With a relatively high work‐function capping layer like ITO over the TiO_x_(h) layer, the band bending is further increased to 0.85 eV, accounting for the emitter formation in *n*‐Si and the highest observed *V*_OC_ of 0.69 V of the Si/TiO_x_(h) solar cell (Figure 3c). A less but non‐negligible band bending (0.35 eV) is induced in *n*‐Si by the TiO_x_(e) deposition, which requires the Si/TiO_x_(e) interface negative charge to reduce to a magnitude below 5 × 10^12^ cm^−2^. This is not an ideal situation because such a band bending repels the photogenerated electrons that come from Si absorber to the electron contact. From the fact that TiO_x_(e) works as an electron‐selective contact in Si solar cell, the influence of negative fixed charge on the electron collection is mitigated by depositing the low work‐function metal capping layer such as Al that reduces the band bending to ≈0.2 eV. However, due to the weakened field‐effect passivation and/or chemical reaction by Al evaporation, the i*V*_OC_ is reduced after Al metallization as shown in Figure 2d. This is a remaining issue that will be addressed in future work to demonstrate higher efficiency solar cells with a symmetrically TiO_x_‐contacted structure.

The presence of negative fixed charge is well known in AlO_x_ (Al_2_O_3_) layers deposited on Si prepared by ALD^[^ ^21^, ^22^, ^23^, ^24^, ^25^ ^]^ and chemical vapor deposition.^[^ ^39^ ^]^ This is also evidenced by measuring the XPS Si 2p peak energy for the Si/AlO_x_ sample in the similar way as done for Si/TiO_x_ (Figure S8, Supporting Information). By exploiting its high negative fixed charge concentration (10^12^–10^13^ cm^−2^), AlO_x_ is widely used in passivating *p* ^+^ emitter and *p*‐Si rear surface, where the negative fixed charges work to repel the minority electrons in Si away from the recombination‐active surface. Despite its widespread use, the microscopic mechanism of the negative fixed charge generation in the Si/AlO_x_ system has not been fully understood. However, the general consensus is that most of the negative fixed charges sit within a first ≈1‐nm‐thick AlO_x_ deposited on Si. As a plausible explanation, the nonstoichiometric growth of AlO_x_ at the beginning of the layer deposition, where O/Al ratio is much higher than in the bulk, is responsible for the change in Al coordination. Specifically, this oxygen‐rich layer is necessary to form a tetrahedrally coordinated Al and to make a chemical bond to the tetrahedrally oriented Si interface, which could be a trigger of negative charge generation.^[^ ^23^ ^]^ The fact that charge generation occurs only when the AlO_x_ is directly deposited on Si indicates that charges can be formed by the chemical reaction between Si and AlO_x_.^[^ ^40^ ^]^

Among various metals, Ti exhibits similar chemical properties to Al, particularly in terms of the large ionization tendency, making them strong reductants. In fact, it is known that titanium oxide is as chemically stable as aluminum oxide. Due to the similar chemical properties, one can expect that the reaction of Si with TiO_x_ also results in a negative fixed charge generation. Similarly to AlO_x_, our in situ XPS measurement elucidates that a high density of negative charges are generated (Si 2p peak shift) within the first ≈1‐nm‐thick TiO_x_ layer on Si. The fact that an almost undetectable SiO_y_ interface layer is grown during the TiO_x_ deposition, indicates that the generation of negative fixed charge is not linked directly to the presence of the SiO_y_ interfacial layer. Rather than this, the growth of metal oxide layer on top of the non‐oxide Si surface is considered as a prerequisite for the generation of negative fixed charge. The difference in electronegativity between metal and Si atoms can lead to a non‐stoichiometric metal oxide growth on Si, which is linked to the fixed charge generation. The source of negative charge is considered as electrons from Si in the case of Si/AlO_x_ system.^[^ ^40^ ^]^ Similarly, electron transfer from Si to Si/TiO_x_ interface is a plausible explanation because most of the charge is generated at the initial stage of the TiO_x_ deposition on Si.

However, in the case of TiO_x_, we found that the amount of fixed charge generation strongly depends on the oxidation route and Ti precursors. In the case of plasma‐ALD TiO_x_, the prior work on capacitance‐voltage and SPV measurements inferred that no significant amount of fixed charges is present at the Si/TiO_x_ interfaces.^[^ ^26^ ^]^ The post‐hydrogenation of plasma‐ALD TiO_x_ increased the hole‐selective nature, as shown in Figure 1, indicating that the hydrogen passivates the interface defects, depinning the Fermi level so that band bending becomes possible by the negative fixed charge. Alternatively, interstitial hydrogen in the SiO_y_ layer could be a possible source of negative charge generation.^[^ ^41^ ^]^ The role of hydrogen in charge generation requires further investigation.

We found that the amount of negative charge generation can be controlled in a thermal ALD process depending on the choice of Ti precursor. We compared two TiO_x_ layers prepared from TTIP and TDMAT using the same oxidant, H_2_O. A marked difference between two Ti precursors is their chemical reactivity with H_2_O (TDMAT ≫TTIP). In fact, a longer H_2_O dose time (3.6 s) by more than two orders of magnitude is necessary for TTIP to have a growth rate saturation in the ALD process than for TDMAT (10 ms). Another difference is the thermal stability of these Ti precursors. Since the thermal decomposition of TDMAT occurs at temperatures of >180 °C,^[^ ^42^ ^]^ we deposited TiO_x_(e) layer at a lower substrate heater temperature (150 °C). On the other hand, the optimum temperature for the TiO_x_(h) using the TTIP is as high as 300 °C. It is considered that the long H_2_O dose per ALD cycle, and relatively high deposition temperature required for the TiO_x_(h) deposition, causes H_2_O or its decomposed molecule to be supplied to the Si/TiO_x_ interface even after a few nanometers of TiO_x_ growth. In fact, as indicated in the in situ XPS measurement (insets of Figure 5a,b), the Si‐O bonding signal increases with increasing TiO_x_ thickness up to 5 nm for the TiO_x_(h) prepared from TTIP, whereas almost no Si‐O peak is observed for electron selective TiO_x_. Therefore, the interfacial chemical reaction at the Si/TiO_x_(h) interface is believed to continue during ALD deposition up to 5 nanometers owing O and H diffusing to the interface, leading to a higher negative fixed charge concentration.

In this study, we used two specific Ti precursors: TTIP and TDMAT. It has been reported that TiCl_4_ is an alternative Ti precursor for thermal ALD that provides excellent surface passivation due to the presence of Cl at the Si/TiO_x_ interface.^[^ ^43^, ^44^ ^]^ It would be worthwhile to apply this precursor in bipolar TiO_x_‐contacted solar cells, as well as in future XPS measurements.

Finally, we discuss the feasibility of bipolar TiO_x_ contacts for industrial Si solar cells. One major advantage of our cell process is that the passivating contact material can be prepared in a single ALD chamber, minimizing equipment costs. However, the current cell process requires two ALD processes for each surface of the Si wafer, which does not fully leverage the benefits of the ALD technique, where thin layers can be deposited on both sides of the wafer in a single deposition run. Therefore, it would be more ideal to deposit the same TiO_x_ layers on the front and rear simultaneously, after which the carrier selectivity of each TiO_x_ layer can be adjusted through postdeposition treatment and the choice of capping electrode. Nevertheless, this has not been successfully demonstrated in our current study, and further research is necessary to achieve this ultimate goal.

## 4 Conclusions

We have studied the material and interface properties of amorphous TiO_x_ layers deposited on *n*‐Si by ALD for application as carrier selective passivating contacts in crystalline Si solar cells. A unique feature of TiO_x_ is the tunable electron/hole selectivity, which is demonstrated here using different oxidation routes (plasma‐ and thermal‐ALD) and Ti precursors (TTIP and TDMAT). In thermal ALD processes, we have successfully synthesized TiO_x_ layers that act as either electron or hole selectivity while providing moderate surface passivation. This allows us to fabricate the world's first symmetrically TiO_x_‐contacted Si solar cells exhibiting ≈19% efficiency. By exploiting ex situ and in situ XPS measurements, we find that the TiO_x_ bulk layer properties are essentially the same regardless of carrier selectivity, and its deposition on Si generates negative fixed charges, as similarly observed in Si/AlO_x_ interfaces. The nature of exhibiting opposite carrier selectivity in a single TiO_x_ material is primarily associated with the different concentrations of negative charge generated at the Si/TiO_x_ interface, which depends on the chemical reaction of Ti precursors and oxidants during growth. The deposition of hole‐selective TiO_x_ creates a hole inversion layer in *n*‐Si with a band bending of 0.7 eV, which is greater than for the case of electron‐selective TiO_x_ (0.35 eV) due to the higher concentrations of negative fixed charges. It is also found that the majority of the negative fixed charges are generated during the first ≈1 nm‐thick TiO_x_ layer deposition (5–10 ALD cycles), while additional charges are generated with thickening the TiO_x_ layer in the case of hole‐selective TiO_x_. The second decisive factor in determining carrier selectivity is the choice of the appropriate capping material for the respective TiO_x_ layers. We found that the use of high‐WF and low‐WF capping electrode is essential for TiO_x_(h) and TiO_x_(e), respectively. Thirdly, post deposition process, such as hydrogen plasma treatment, can increase the hole selectivity of TiO_x_ (while reducing its electron selectivity). Meanwhile, the H‐containing SiO_y_ interfacial layer is predominantly grown by post‐oxidation during the annealing, which is found to increase the chemical passivation in both hole‐ and electron‐selective TiO_x_ layers. The bipolar TiO_x_ passivating contacts offer versatility, with potential uses in various high‐efficiency Si‐based solar cell architectures.

## Conflict of Interest

The authors declare no conflict of interest.

## Author Contributions

T.M. supervised the project, designed the experiments, performed the ALD processes, thin‐film coatings, XPS measurements at AIST, and analyzed the experimental data. S.F., K.G., and N.U. developed the ALD processes for electron‐selective TiO_x_ layers using a TDMAT precursor at Nagoya University. S.F. performed QSSPC, PL, and contact resistivity measurements at AIST, and analyzed the experimental data. H.S. constructed the baseline processes and characterizations for crystalline Si solar cells at AIST. S.M. and R.S.B. conducted and analyzed G−V, KP, and XPS measurements at University of Oxford. S.M. performed the spectral fitting for the XPS spectra. J.M. and R.S.B. carried out device simulations. T.M., S.M., and R.S.B. wrote the manuscript. All authors discussed the results and provided comments on the manuscript.

## Supporting information



Supporting Information

## Data Availability

The data that support the findings of this study are available from the corresponding author upon reasonable request.
