# Supplementary material for: Symmetric Dopant‐Free Si Solar Cells Enabled by TiOx Nanolayers: An In‐Depth Study on Bipolar Carrier Selectivity
Source: Adv Sci (Weinh). 2024 Nov 28;12(3):2410179. doi: 10.1002/advs.202410179 (PMC11744698; doi:10.1002/advs.202410179)
Supplement: Supplementary file 1 — Supporting Information [file ADVS-12-2410179-s001.pdf]

ADVANCED SCIENCE
Open Access

Supporting Information

for *Adv. Sci.*, DOI 10.1002/advs.202410179

Symmetric Dopant-Free Si Solar Cells Enabled by TiO_x Nanolayers: An In-Depth Study on Bipolar Carrier Selectivity

Takuya Matsui, Shohei Fukaya, Shona McNab, James McQueen, Kazuhiro Gotoh, Hitoshi Sai, Noritaka Usami and Ruy Sebastian Bonilla*

Supporting Information

Symmetric Dopant-Free Si Solar Cells Enabled by TiO_x Nanolayers: An In-Depth Study on Bipolar Carrier Selectivity

Takuya Matsui,[1,2] Shohei Fukaya,[1,2] Shona McNab,[3] James McQueen,[3] Kazuhiro Gotoh,[2] Hitoshi Sai,[1] Noritaka Usami,[2] and Ruy Sebastian Bonilla[3]*

[1]Renewable Energy Research Center, National Institute of Advanced Industrial Science and Technology (AIST), 1-1-1 Umezono, Tsukuba, Ibaraki, 305-8568, Japan
[2]Graduate School of Engineering, Nagoya University, Furo-cho, Chikusa-ku, Nagoya 464-8603, Japan
[3]Department of Materials, University of Oxford, Parks Rd, Oxford, OX1 3PH, United Kingdom
E-mail: t-matsui@aist.go.jp

Experimental Procedures:

ALD and HPT Processes

TiO_x layers were deposited by three ALD systems. Most of the TiO_x(h) and TiO_x(e) layers (except for TiO_x layers presented in Figure 1, 5 and 6) including those for symmetrically TiO_x-contacted solar cells were prepared in a custom-made ALD chamber equipped with an inductively-coupled plasma source and a load lock chamber (Eiko Corporation). In this ALD system, all TiO_x layers were grown by thermal ALD using H_2O as an oxidant. TiO_x(h) layers were grown at a substrate heater temperature of 300 °C using a TTIP precursor. TTIP dose time was 1.2 s. The H_2O dose was controlled by the valve opening time (1.2 s) and the number of H_2O pulses (3 times) in an ALD cycle. By repeating ALD cycle for 128 times, ~5 nm-thick TiO_x(h) layer was deposited. Then, TiO_x(h) layer underwent a hydrogen plasma treatment (HPT) in the same ALD chamber under a pressure of 10 Pa, a H_2 flow rate of 100 SCCM and an rf plasma power of 600 W. TiO_x(e) layers were grown in the same ALD chamber but using a TDMAT precursor at a substrate heater temperature of 150 °C. The H_2O dose time was 10 ms. By repeating ALD cycle for 80 times, ~5 nm-thick TiO_x(e) layer was deposited. TTIP and TDMAT source temperature was kept at 60 °C in a constant temperature bath and they were delivered to the chamber by an Ar bubbler system.

For in situ ALD-XPS experiments presented in Figure 5 and 6, a different ALD system (FlexAL, Oxford Instruments) was used, which is connected with an XPS analytic chamber via a high-vacuum transfer chamber. For TiO_x(h) and TiO_x(e) layers deposition, almost the same ALD conditions were used as used for solar cell fabrication, except some minor differences depending on the chamber design, resulting in reproducible growth rate per cycle.

For TiO_x layers presented in Figure 1, another ALD system (FlexAL, Oxford Instruments) was used for both thermal- and plasma-ALD processes, which is the same as that use in the previous work.[1] TiO_x layers were deposited from the TTIP precursor at a substrate heater temperature of 240 °C.

Fabrication of Test Samples and Solar Cells

In this study, *n*- and *p*-type float zone monocrystalline Si wafers (phosphorous-doped *n*-type or boron-doped *p*-type Si, 2-3 Ωcm, (100) orientation, 280 μm thick) were used. These Si wafers were cleaned using H_2SO_4, H_2O_2, HCl, and HF solutions.[2] A short dip of wafers in a diluted HF solution was done to strip native oxide prior to any layer deposition. For test samples (Figure 1 and 2), DSP Si wafers were used. One side of the wafer was capped with a-

Si:H layer(s) deposited by plasma-enhanced chemical vapor deposition used for SHJ solar cells.[2] After an additional HF-dip, TiO_x layers were deposited on another side of the Si wafers.

For symmetrically TiO_x-contacted solar cells, the DSP and FT_RP *n*-Si were used. The latter Si substrates were prepared in a same manner as reported in Ref. [3]. After HF-dip, either TiO_x(h) or TiO_x(e) layer was deposited by ALD on one side of the *n*-Si substrate. Then, the *n*-Si wafers were taken out of the vacuum and flipped in the air, and then they were immediately loaded back in the same ALD chamber. Afterwards, the other carrier-selective TiO_x layer was deposited. It should be noted that the ALD sequence (TiO_x(e) -> TiO_x(h) or TiO_x(h) -> TiO_x(e)) does not have a significant impact on the solar cell performance. The TiO_x(h) layer was subjected to HPT for 60 min. Since HPT was done without a substrate rf bias,[3] it leads to longer required treatment time. After these processes in the ALD chamber, a positive front electrode (ITO/Ag-grid, area: 1.04 cm^2) and a negative (LiF/Al, full area) electrode were formed on the TiO_x(h) and TiO_x(e) layers, respectively. Postdeposition annealing was carried out at a temperature of 180 °C in a low-vacuum oven (~10 Pa of air) to enhance the passivation properties at both TiO_x/Si interfaces. Note that this postdeposition annealing was done before the rear electrode formation because the annealing of the Si/TiO_x/LiF/Al stack results in poorer passivation quality probably due to the excessive chemical reaction of TiO_x and Al layers.

Characterizations

The layer thickness of the deposited TiO_x layers was characterized by ex situ spectroscopic ellipsometry (J.A. Woollam). The effective minority carrier lifetime and the iV_{OC} of the solar cell precursors (before metallization) were monitored at each process step with a QSSPC measurement setup (Sinton Instruments, WCT-120). For the finished cells, the $J-V$ characteristics were evaluated at 25 °C using a calibrated dual-light source class AAA WACOM sun simulator with an air mass 1.5 global irradiance spectrum at 100 mW cm^{-2}. The illumination area of 1.045 cm^2 was designated by using a black shadow mask whose aperture area was designed to be slightly smaller than that of the electrode of the emitter side. External quantum efficiency (EQE) spectra were measured with a Bunkou-Keiki, CEP-97 setup. The PL measurement was carried out in an ITES PVX1000+POPLI-Λ system. The samples were homogeneously illuminated by an excitation laser with a wavelength of 850 nm through a beam expander and a PL signal was recorded in a Si-CCD camera with a cut-on wavelength of 990 nm.

HAADF-STEM and EDX measurements were carried out at JFE Techno-Research Corporation. Cross-sections of the samples were observed by STEM imaging using a high-

angle annular dark-field detector combined with EDX spectroscopy at an acceleration voltage of 80 kV (JEOL JEM-ARM200F). To avoid the electron-beam induced degradation of the samples, the EDX scanning was carried out under the minimum irradiation condition. RBS and ERDA measurements were carried out at Kobelco Corporation using a HRBS500 system. RBS measurements were conducted using He^+ ion operated at an acceleration energy of 450 keV with an incident angle of 55° with respect to the Si normal. ERDA measurements were conducted using the N^+ ions operated at an acceleration energy of 480 keV with an incident angle of 70° with respect to the Si normal.

XPS measurements were carried out using two Phi Versaprobe III (PhiV, Ulvac PHI) systems in AIST and at the University of Oxford. In AIST, both ex situ and in situ XPS measurement were done in a PhiV system which is connected to the ALD and load lock chambers via a transfer robot chamber. The PhiV uses a monochromatic A1 Kα X-ray (1.487 keV) source with a 7.5 μm beam diameter, a beam angle of ~90°, and an electron take-off angle of 45°. The lowest pass energy of 6.5 eV is used for the ex situ measurement, while a higher pass energy of 55 eV was used for the in situ measurement, with an intention mentioned in the main text. The Au 1f peak at 83.96 eV was used to calibrate the binding energy. No neutralizer was used during the measurement due to the conductive nature of the TiO_x sample. To ensure adequate charge extraction, sample rear and front were contacted by a metal plate and clamp, respectively. Peak fitting was performed in CasaXPS using Shirley backgrounds and pseudo-Voigt (LA50) line shapes. Ti 2p and Si 2p have closely spaced spin-orbit components, which was considered for fitting the Si^0 and Ti^0 where this could be well resolved, but was not considered for the other components. Savitzky–Golay linear smoothing over 5 data points was applied to the raw data after the peak fitting operations to aid visual inspection of the data.

$G-V$ and KP measurements were conducted in University of Oxford. The details of measurement setups and conditions can be found in Ref. [3].

References:

[1] T. Matsui, M. Bivour, M. Hermle, H. Sai, *ACS Appl. Mater. Interfaces* **2020**, *12*, 49777−49785.

[2] H. Sai, T. Oku, Y. Sato, M. Tanabe, T. Matsui, K. Matsubara, *Prog. Photovolt. Res. Appl.* **2019**, *27*, 1061-1070.

[3] T. Matsui, S. McNab, R. S. Bonilla, H. Sai, *ACS Appl. Energy Mater.* **2022**, *5*, 12782−12789.

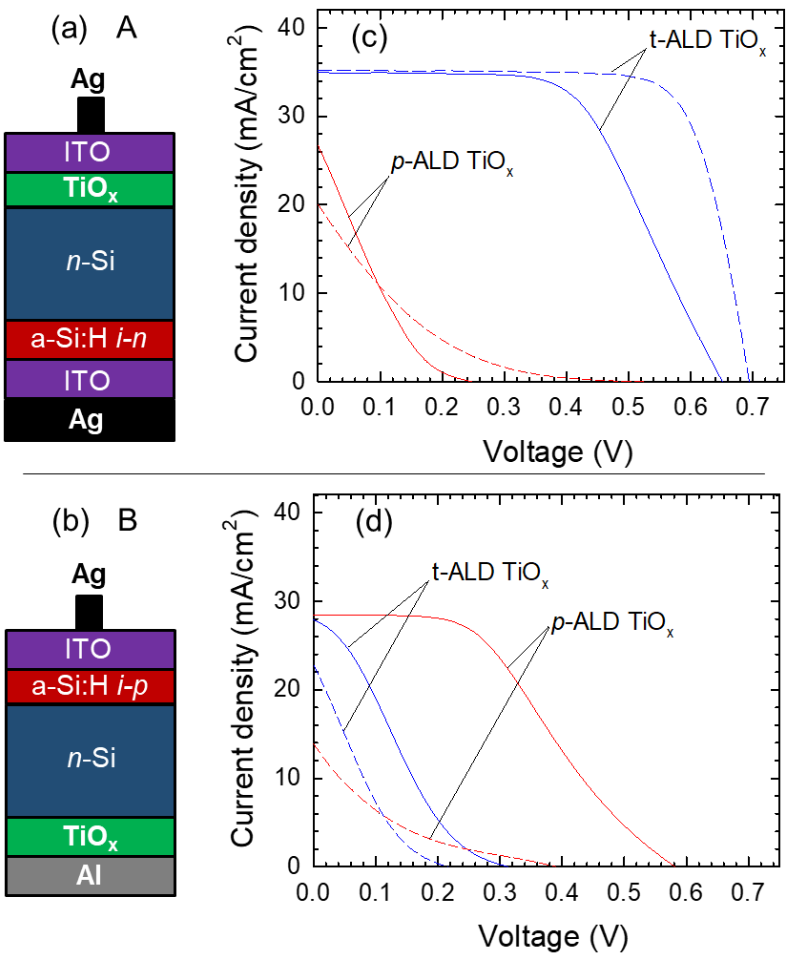

Figure S1. (a, b) Test solar cell structures of (a) device A and (b) device B that have *n*- and *p*-doped a-Si:H layers for evaluating hole and electron selectivity of the TiO_x layers, respectively. (c, d) Illuminated $J-V$ curves of the test solar cells with TiO_x layers made by thermal-ALD (blue) and plasma-ALD (red) in the initial state (solid lines) and after hydrogen plasma treatment followed by thermal annealing at 180 °C (dashed lines). The results obtained using the devices A and B correspond to (c) and (d), respectively.

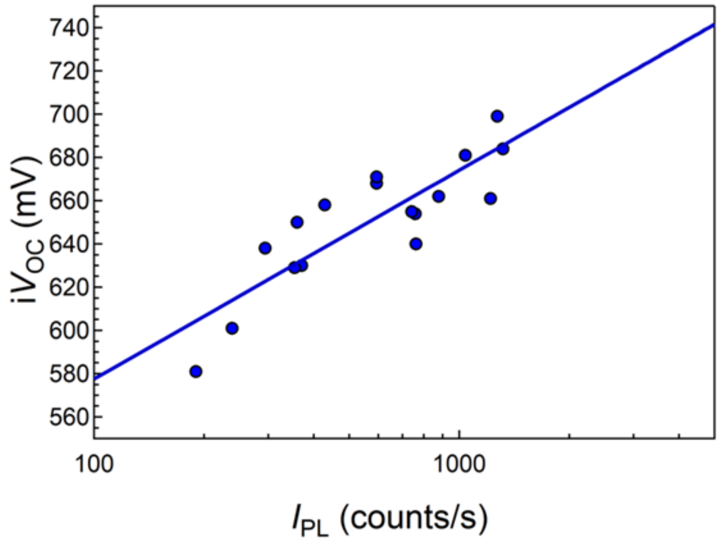

Figure S2. Correlation between iV_{OC} measured by QSSPC and PL intensity I_{PL} (circles) of the asymmetrically passivated sample structure (TiO_x/*n*-Si (100)/a-Si:H). The line represents a linear fit to the measured data, which is used as a calibration line for estimating the iV_{OC_PL} of the metallized samples.

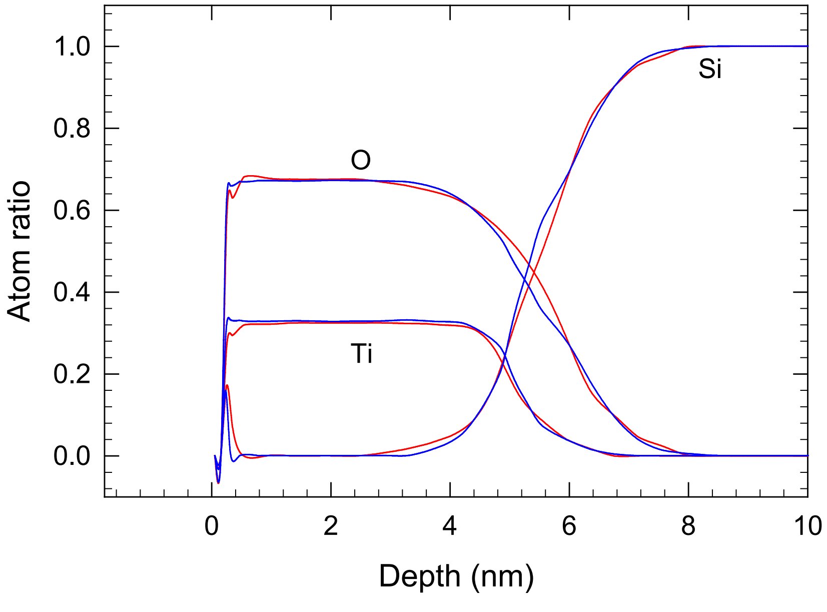

Figure S3. Elemental depth profiles near the Si/TiO_x(h) (blue) and Si/TiO_x(e) (red) interfaces for Ti, O and Si measured by RBS. Thickness of the TiO_x layers was determined by the spectroscopic ellipsometry. The RBS measurements were performed without capping layer deposition nor annealing. It can be seen that the atomic compositions in the TiO_x bulk layers are essentially the same while the O/Si ratio at the Si/TiO_x interface is slightly higher for the TiO_x(e) than for the TiO_x(h), in agreement with the ex situ XPS results shown in Figure 4a.

Table S1. Film density and the average atomic compositions of the TiO_x(h) and TiO_x(e) bulk layers measured by RBS for Ti and O, and ERDA for H.

sample	film density (g cm^{-3})	Ti (at.%)	O (at.%)	H (at.%)	O/Ti
TiO_x(h) (TTIP)	3.0	32.4	66.2	1.4	2.04
TiO_x(e) (TDMAT)	2.4	29.2	60.8	9.9	2.08

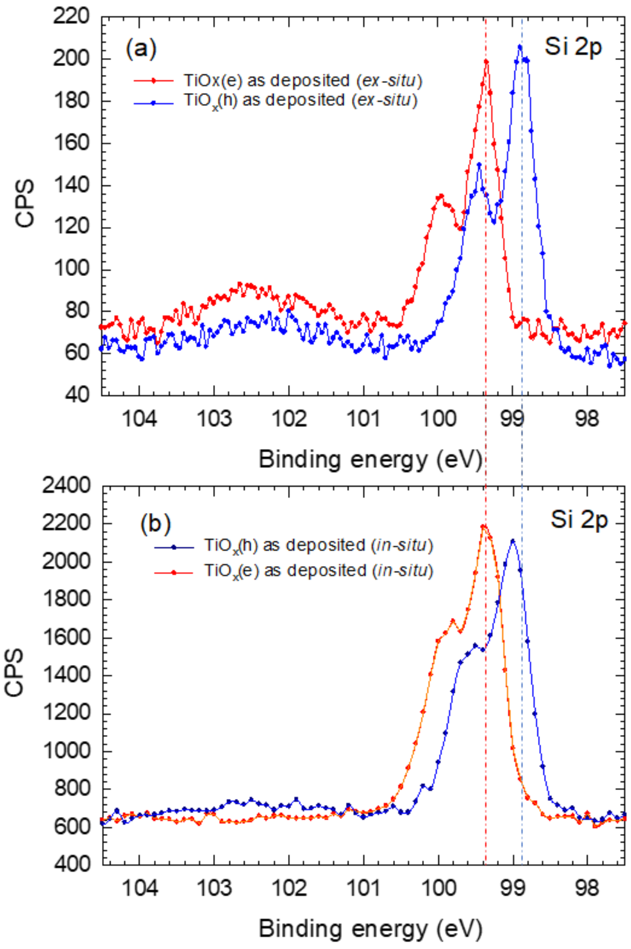

Figure S4. Comparison of XPS Si 2p spectra of *n*-Si/TiO_x(h) (blue) and *n*-Si/TiO_x(e) (red) samples measured in (a) ex situ and (b) in situ after depositing ~5-nm-thick TiO_x layers. TiO_x layers for ex situ and in situ XPS measurements were grown in two different ALD systems while the measurements were carried out in the same XPS setup. Thus, the TiO_x layers for ex situ measurement were subjected to an air break. Note that the lower pass energy was used for the in situ measurement, resulting in the higher signal intensity and lower spectral resolution. Although the Si 2p peaks of the TiO_x(h) samples differ by 0.1 eV between those measured by ex situ and in situ XPS, the peak shift with respect to those of TiO_x(e) layers can still be evaluated. In (b), it is found that almost no SiO_y interfacial layers is formed during the TiO_x deposition particularly for the TiO_x(e) sample. This indicates that SiO_y interface layer is predominantly formed by the oxygen supply from the ambient air.

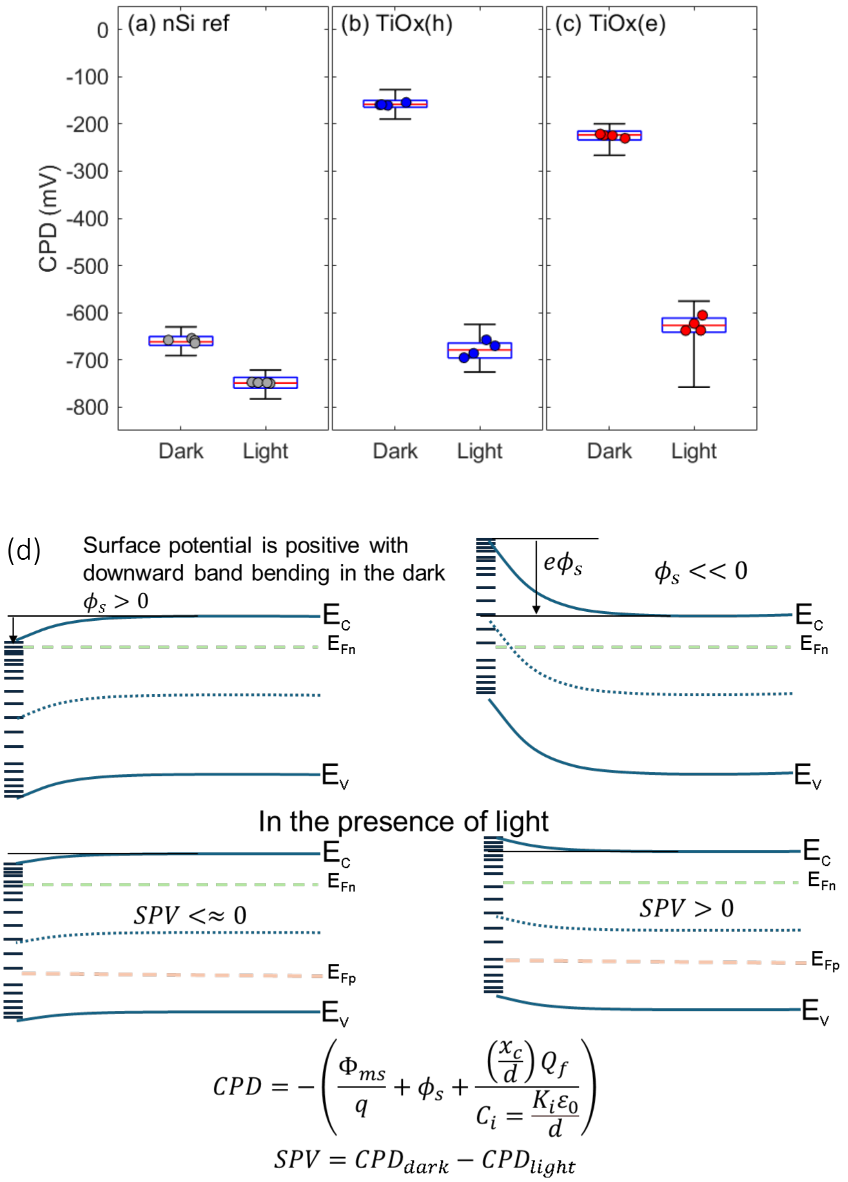

Figure S5. Surface photovoltage (SPV) contact potential difference (CPD) measurements using Kelvin probe (KP) on (a) *n*-Si alone, (b) *n*-Si/TiO_x(h) and (c) *n*-Si/TiO_x(e), showing a negative value of SPV for both *n*-Si/TiO_x(h) and *n*-Si/TiO_x(e) samples. However, CPD signal of the TiO_x(h) is higher than the TiO_x(e), indicating that TiO_x(h) has more negative fixed charges. (d) Schematic representation of CPD measured in the KP instrument, and the extracted SPV linking to the polarity of charge. Where, Φ_{ms} is the work function difference between the gold probe and the silicon, q is the electron charge, ϕ_s is the surface potential, x_c is the distance of the charge centroid from the interface, d is the TiO_x thickness, Q_f is the fixed charge density, C_i is the capacitance generated from the interface defects and K_i is the TiO_x dielectric constant and ε_0 is the permittivity of free space.

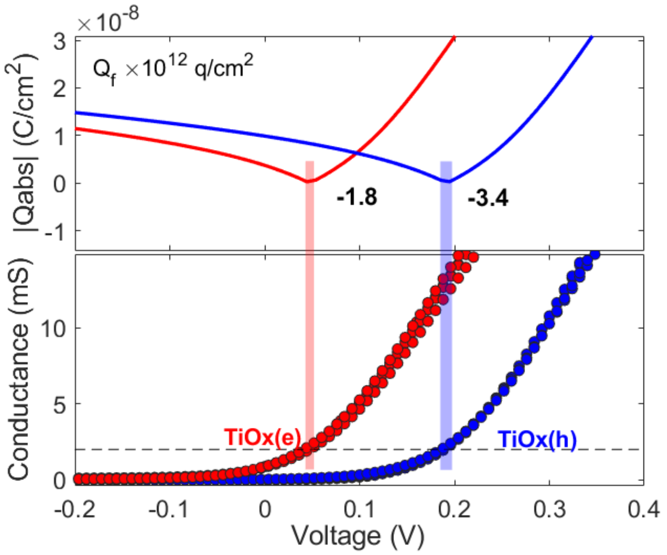

Figure S6. (a) Conductance−voltage (*G−V*) measurements of metal/*n*-Si/TiO_x/Al structures, for TiO_x(h) (blue line and symbols) and TiO_x(e) (red line and symbols), using planar (100) 2 Ωcm *n*-Si, and including modelled dependence of the surface charge concentration as a function of the metal and internal dielectric-Si interface charge.

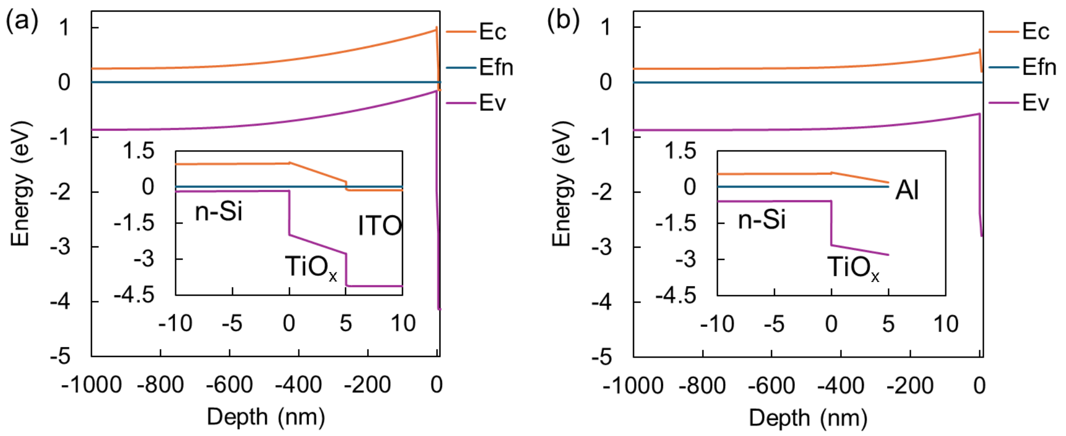

Figure S7. Finite element device simulations of (a) *n*-Si/TiO_x(h)/ITO with interface charge of Q_f~-1×10^{13} q/cm^2 resulting in ~0.86 eV band bending. (b) *n*-Si/TiO_x(e)/Al with interface charge Q_f~-5×10^{12} q/cm^2 resulting in ~0.2 eV band bending. Simulations were conducted in SCAPS (scaps.elis.ugent.be) and the material parameters used are listed in Table S2.

Table S2. Material parameters assumed in the SCAPS simulations.

Material parameters	Si	TiO_x(h)	TiO_x(e)	ITO	Al
Bandgap (eV)	1.12	3.5	3.5	4.0	-
Electron affinity (eV)	4.05	4.0	4.0	4.6	4.2
Relative permittivity	11.9	15.0	15.0	9.0	-
Electron concentration (cm^{-3})	1.5×10^{15}	1.0×10^{13}	1.0×10^{13}	1.0×10^{20}	-

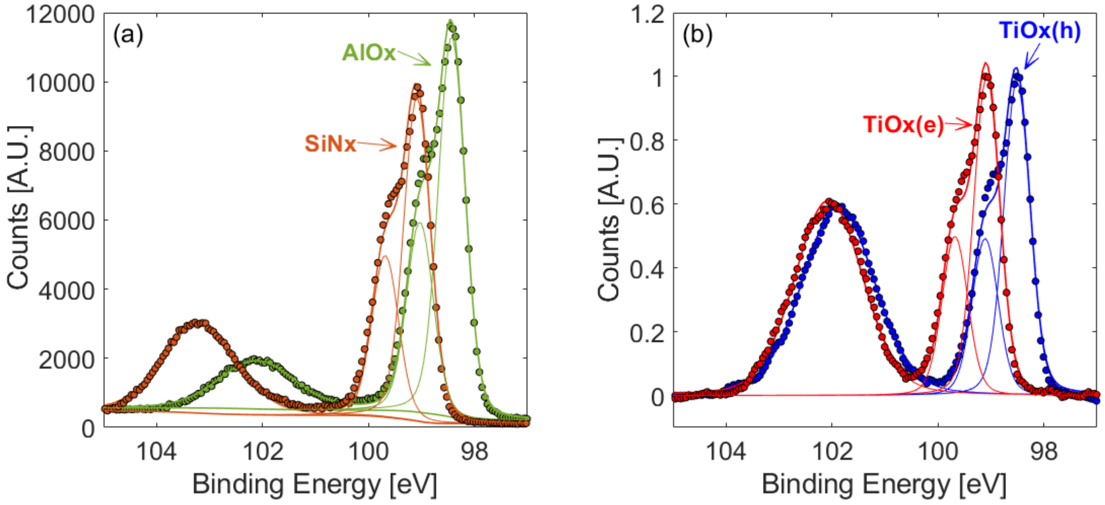

Figure S8. (a) An example of XPS Si 2p spectra for *p*-Si (100)/1.5 nm AlO_x (green symbols and lines) and *p*-Si (100)/2 nm SiN_x (orange symbols and lines) samples. (b) XPS measurements of the Si 2p peak of *n*-Si (100)/TiO_x(h) (blue symbols and lines) and *n*-Si (100)/TiO_x(e) (red symbols and lines) layers after PDT. These measurements were taken in University of Oxford using a PhiVersaprobeIII XPS system with a monochromatic A1 Kα X-ray (1.487 keV) source with a 100 μm beam diameter, a beam angle of ~90°, and an electron take-off angle of 45°. A pass energy of 20 eV was used and the carbon 1s peak at 284.7 eV was used to calibrate the samples from any surface charge generated at the sample surface due to the X-ray beam. The XPS spectra shown in (b) measured at University of Oxford is in agreement with those measured at AIST shown in Figure 4b.
